# Simvastatin dose and acute kidney injury without concurrent serious muscle injury: A nationwide nested case-control study

**DOI:** 10.1371/journal.pone.0182066

**Published:** 2017-07-28

**Authors:** Lianne Parkin, Katrina J. Sharples, David J. Barson, Mei-Ling Blank

**Affiliations:** 1 Department of Preventive and Social Medicine, Dunedin School of Medicine, Division of Health Sciences, University of Otago, Dunedin, New Zealand; 2 Department of Mathematics and Statistics, Division of Sciences, University of Otago, Dunedin, New Zealand; 3 Department of Medicine, Dunedin School of Medicine, Division of Health Sciences, University of Otago, Dunedin, New Zealand; University of British Columbia, CANADA

## Abstract

**Background:**

Inconsistent findings from four observational studies suggest that the risk of acute kidney injury (AKI) may increase with increasing statin dose or potency, but none of the studies took statin-related severe muscle injury, including rhabdomyolysis, into account. We undertook a nationwide nested case-control study in New Zealand to examine the risk of AKI without concurrent serious muscle injury according to simvastatin dose in two cohorts: people without a history of renal disease and people with non-dialysis dependent chronic kidney disease.

**Materials and methods:**

A total of 334,710 people aged ≥ 18 years without a history of renal disease (cohort 1) and 5,437 with non-dialysis dependent chronic kidney disease (cohort 2) who initiated simvastatin therapy between 1 January 2006 and 31 December 2013 were identified using national pharmaceutical dispensing and hospital discharge data. Patients who developed AKI without concurrent serious muscle injury during follow-up (cases) were ascertained using hospital discharge and mortality data (n = 931 from cohort 1, n = 160 from cohort 2). Up to 10 controls per case, matched by date of birth, sex, and cohort entry date were randomly selected from the relevant cohort using risk set sampling.

**Results:**

Relative to current use of 20mg simvastatin daily, the adjusted odds ratios and 95% confidence intervals (95% CI) in cohort 1 for current use of 40mg and 80mg were 0.9 (95% CI 0.7–1.2) and 1.3 (95% CI 0.7–2.3), respectively. The adjusted odds ratio for 40mg in cohort 2 was 1.1 (95% CI 0.7–1.9); the numbers taking 80mg were very small and the confidence interval was correspondingly wide.

**Conclusion:**

The findings of this study suggest that a relationship between statin dose and AKI may not exist independent of serious muscle injury.

## Introduction

Statins play a key role in the prevention of major cardiovascular events [1–5]. The introduction of preventive strategies which consider the combined effects of multiple risk factors has led to the widespread use of these drugs, and evidence of additional benefit with intensive therapy has led to the prescription of higher doses [5, 6]. With the growing use of statins by people with low to moderate cardiovascular risk, reliable estimates of potential adverse effects have become increasingly important [6–8].

Four observational studies have recently explored the relationship between statin use and the risk of acute kidney injury (AKI) and the findings are mixed [9–12]. Three found a higher risk of AKI in users of high versus low potency statins [10–12], although in one study the association was confined to patients with no pre-existing renal disease and was not observed in those with non-dialysis dependent chronic kidney disease [10]. Conversely, in a later study, no association was found in a healthy cohort without chronic renal disease [12]. Only one study explored the risk of AKI in users of high versus low doses of individual statin drugs and although the findings were suggestive of a dose-response relationship for some statins, chance could not be excluded as a potential explanation [9]. Importantly, AKI occurs with rhabdomyolysis and the risk of rhabdomyolysis increases with increasing statin dose [6] [13], but none of the observational studies systematically excluded patients with serious muscle injury (severe myopathy, including rhabdomyolysis). Information from randomised controlled trials is very limited; most statin trials have been placebo-controlled and those which did assess outcomes in users of high versus low statin doses or potency were not powered to detect a difference in the occurrence of AKI [14–19]. Hence, it remains unclear whether statin use increases the risk of AKI independent of serious muscle injury [6] [13].

We undertook a nationwide study to examine the risk of AKI in relation to the dose of simvastatin (the first-line statin in New Zealand during the study period), explicitly excluding patients who developed AKI in conjunction with a muscle disorder that was serious enough to result in a hospital diagnosis or death. We explored the risk of AKI in case-control analyses nested within two cohorts of new users of simvastatin: those with no history of renal disease and those with non-dialysis dependent chronic kidney disease.

## Materials and methods

### Data sources

The study was based on administrative health and pharmaceutical dispensing data provided by the New Zealand Ministry of Health. As previously described [20], the Ministry holds several national data collections including the National Minimum Dataset (public and private hospital admissions), the Mortality Collection (hospital and community-based deaths), the New Zealand Cancer Registry (all cancers, except non-melanoma skin cancers), and the Pharmaceutical Collection (records of all claims by community-based pharmacists for the dispensing of prescription drugs which are publicly funded) [21–24]. Individual patient records in these data collections are indexed to a unique alpha-numeric identifier, the National Health Index (NHI) [25], which enables the linkage of patient-level health and pharmaceutical dispensing data, along with the demographic data held in the NHI Collection. Diagnoses in the National Minimum Dataset, Mortality Collection, and Cancer Registry are coded to successive revisions of the International Statistical Classification of Diseases and Related Health Problems, Australian Modification, (ICD-AM) and procedures in the National Minimum Dataset are coded to the Australian Classification of Health Interventions (ACHI) [26].

The Ministry of Health identified all patients who were dispensed simvastatin (including simvastatin in combination with ezetimibe) at least once in New Zealand between 1 January 2005 and 31 December 2013 (*the provisional cohort*). Cohort entry was defined as the date of the first dispensing of simvastatin during that period; the end date of follow-up was the earliest of a diagnosis of AKI, death, or 31 December 2013. For each patient, the Ministry provided the following information: demographic data (sex, date of birth, date of death, prioritised ethnicity [27], and an area-based measure of socioeconomic position, NZDep06 [28]); details of all dispensings of simvastatin and other medicines (including subsequent dispensings of other statins) from cohort entry; details of any hospital admissions before (from 1988 for public hospitals) and after cohort entry; details of any cancer registrations before and after cohort entry; and details of any deaths. The data were supplied with encrypted NHIs, except for patients who were admitted to hospital and/or died after cohort entry with an ICD-10-AM diagnosis of N17 (Acute renal failure) for whom unencrypted NHIs were provided.

### Identification of the two study cohorts

**Fig 1:**
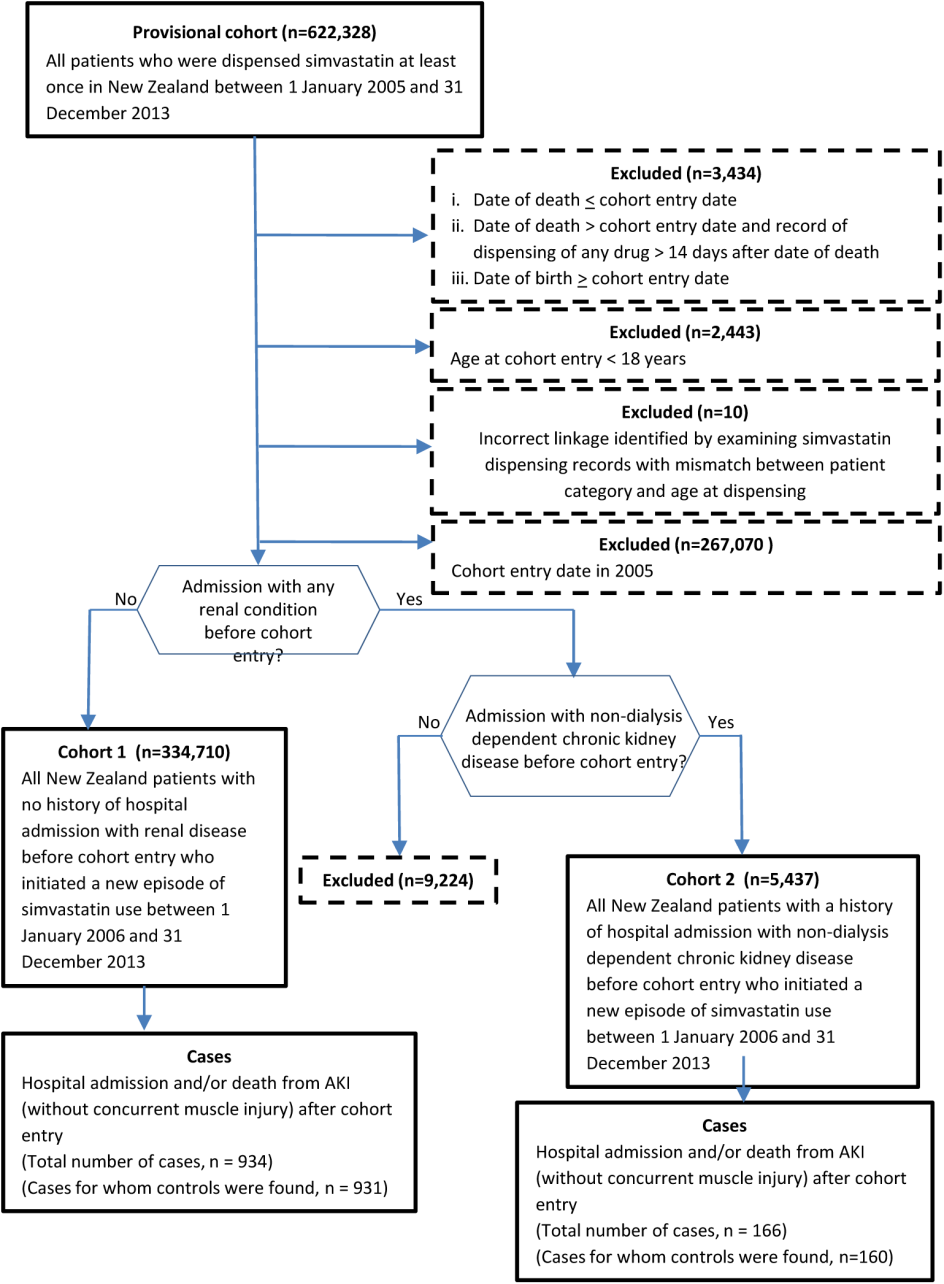
Identification of the two study cohorts and cases.

Several steps were undertaken to derive the two study cohorts from the provisional cohort (Fig 1). First, we excluded any linked records for which the dispensing information and the health data clearly did not refer to the same person. Second, we excluded all provisional cohort members with a cohort entry date between 1 January and 31 December 2005. This was to ensure that cohort members were followed from the initiation of simvastatin therapy (either as naïve users or past users who were restarting after a break of at least one year). Third, we divided the remaining provisional cohort members into two groups: those who did, and those who did not (cohort 1), have at least one hospital admission with a diagnosis (coded as a “principal diagnosis” or “additional diagnosis” [29]) of a renal condition before their respective cohort entry dates. The relevant ICD-9-AM and ICD-10-AM renal diagnosis codes, which are based on those we used in a previous investigation [30], are shown in S1 Appendix. Finally, to enable a comparison with the results of a recent study [10], the group with a history of renal disease at cohort entry was restricted to those with non-dialysis dependent chronic kidney disease (cohort 2) as per the criteria in S2 Appendix.

### Nested case-control analyses

The methods for the nested case-control analyses in cohort 1 and cohort 2 were identical and are outlined below. Identification of the cases and selection of the controls was undertaken independently of information about prescribed doses of simvastatin

#### Identification of cases

Hospital discharge and mortality data were searched to identify cohort members who developed AKI without serious muscle injury between cohort entry and 31 December 2013. Patients were classified as cases if (i) there was a record of a hospital admission in which there was a principal diagnosis of N17 and in the same admission there were no additional diagnoses coded to the muscle-related ICD-10-AM rubrics under which rhabdomyolysis may be classified (see S3 Appendix); (ii) N17 was recorded as the underlying cause of death (for deaths between cohort entry and 31 December 2011); or (iii) the Cause of Death free text fields [31] in the Mortality data (for deaths in 2012 and 2013 which had not yet been coded) indicated that AKI was the underlying cause of death and there was no mention of rhabdomyolysis or myopathy. Cases were defined as having undergone dialysis if any of the dialysis-related ICD-10-AM and ACHI codes listed in S2 Appendix were recorded immediately before or during the case-defining admission. The date of the hospital admission (or death if no admission) in which AKI was diagnosed was taken as the index date for the cases and their matched controls. For cases who underwent dialysis, the index date was the earlier of the date of admission with AKI and the date of first dialysis.

#### Selection of controls

Up to 10 controls per case, matched by date of birth (+/- 183 days), sex, and cohort entry date (+/- 183 days), were randomly selected from the relevant study cohort using risk set sampling [32]. As with cases, patients who had a hospital admission between cohort entry and the index date in which N17 was the principal diagnosis and there were additional muscle-related diagnoses were not eligible for inclusion.

#### Simvastatin exposure

The impact of two different definitions of exposure were explored in the analyses: “as treated exposure” and “fixed exposure”. In the “as treated” definition, cases and controls were classified as current users of simvastatin if their dispensed supply extended into the 30 day period before the index date. Current users were further categorised according to their daily dose of simvastatin. Recent and past users were those whose dispensed supply of simvastatin terminated within 31–90 days and > 90 days before the index date, respectively. Conversely, in the “fixed exposure” definition, cases and controls were classified as users of the daily dose of simvastatin they were dispensed on their cohort entry date.

#### Other key variables

The following information was extracted from the health and dispensing records of cases and their matched controls: demographic characteristics (ethnicity, NZDep06 score); a history before cohort entry of admissions with conditions which might have influenced prescribing decisions about simvastatin dose (including type 1 diabetes, type 2 diabetes, raised blood pressure, dyslipidaemia, tobacco use, obesity, myocardial infarction, angina, coronary artery bypass graft, percutaneous coronary intervention, congestive heart failure, ischaemic stroke, transient ischaemic attack, atrial fibrillation, and peripheral arterial disease) [33]; a history before the index date of admissions with comorbidities postulated to increase the risk of AKI in general (including major trauma, major surgery, sepsis, shock, type 1 diabetes, type 2 diabetes, myocardial infarction, angina, acute heart failure, chronic liver disease, and cancer) [34] [35]; and the use of other drugs before the index date (excluding topical preparations) which are known or suspected to cause AKI (including acyclovir, aminoglycosides, amphotericin, angiotensin converting enzyme inhibitors, angiotensin receptor blockers, β-lactam antibiotics, chemotherapy, cisplatin, diuretics, methotrexate, non-steroidal anti-inflammatory drugs (NSAIDs), radio-contrast agents, sulphonamides, and tacrolimus) [34] [36] or to interact with statins (including amiodarone, azol antifungals, calcium channel blockers, cyclosporin, danazol, fibrates, fusidic acid, macrolide antibiotics, nefazodone, nicotinic acid, protease inhibitors, thiazolidinediones/glitazones, and warfarin) [37–39]. A Charlson comorbidity score at cohort entry was also derived for each case and control, based on hospital admissions in the preceding five years, using an adapted version of an ICD-10 SAS macro developed by others [40].

#### Statistical methods

The primary analysis explored the risk of AKI without serious muscle injury according to daily dose of simvastatin in current users, with current use of 20mg as the reference category (“as-treated exposure” analysis). Recent and past users were treated as separate categories in the analysis (relative to the reference category). Secondary analyses explored the relationship between simvastatin dose and AKI without serious muscle injury (i) in an analysis confined to the sub-group of cases who required dialysis and their controls (“as-treated exposure” analysis) and (ii) according to the dose of simvastatin which was dispensed at cohort entry (“fixed exposure” analysis). Conditional logistic regression was used to estimate odds ratios (ORs) and 95% confidence intervals (95% CIs), and to explore potential confounding by ethnicity, socioeconomic position, and the comorbidities and drugs listed above. Model selection was undertaken using a backwards stepwise elimination process. The variables included initially were: ethnicity (Māori, Pacific, Asian and European or Other), NZDep06 quintile, smoking (ever recorded in hospital discharge data or dispensed smoking cessation medications in the year before the index date), obesity (ever recorded in hospital discharge data or dispensed weight loss drugs in the year before the index date), diabetes mellitus (any type of diabetes mellitus ever recorded in hospital discharge data or dispensed insulin or oral hypoglycaemics in the year before the index date), Charlson comorbidity score, age at cohort entry (to adjust for any residual confounding by age), indicator variables for conditions (before cohort entry) which might have influenced prescribing decisions about simvastatin dose, indicator variables for conditions which might increase the risk of AKI (before cohort entry, and between cohort entry and the index date), the number of hospital admissions for any reason in the year before cohort entry, the number of hospital admissions for any reason in the year before the index date, and indicator variables for the dispensing of drugs which are associated with AKI and/or may interact with statins. Variables for simvastatin use, ethnicity, NZDep06, and Charlson comorbidity score were retained in the model without consideration of p-values. The remaining possible confounders were retained where p < 0.2. Missing data on ethnicity and NZDep were imputed using multiple imputation with chained equations (50 imputations) [41], with imputations based on all model covariates plus case-control status. Analyses were carried out in Stata version 13.1 [42].

### Estimation of incidence rates in cohorts 1 and 2

The simvastatin dispensing data of each member of the respective study cohorts were summarised into continuous episodes of use; a continuous episode was defined as one in which the elapsed time between the end date of one dispensed supply and the start date of the next was 30 days or less. Episodes were censored at the earliest of the following: another statin was dispensed, the patient died, the study period ended (31 December 2013), or the index date was reached (if a case). The durations of episodes were added together to obtain total person-years of exposure for the two cohorts separately. The incidence rate of AKI without serious muscle injury in each cohort was estimated by dividing the number of cases who were current users on the index date by the total person-years of exposure; 95% CIs were calculated using Epi Info Version 3.01 (Mid-P exact test) [43].

### Review of spontaneous reports of adverse reactions

In a separate investigation, the database of the Centre for Adverse Reactions Monitoring (CARM) at the New Zealand Pharmacovigilance Centre was searched to determine the proportion of our cases who had been reported to CARM and, conversely, whether there were any reported cases of AKI without serious muscle injury who met our eligibility criteria but were not identified and included in our study. New Zealand Pharmacovigilance staff used the NHI, date of birth, sex, index date, and cohort entry date as linkage keys and provided us with anonymised data for this part of our study.

### Ethical approval

The study was approved by the Northern A Health and Disability Ethics Committee (reference: HDEC/14/NTA/178). The study was based on routinely collected summary data and no patient consent was required.

## Results

The Ministry of Health identified a provisional cohort of 622,328 people who were dispensed simvastatin at least once between 1 January 2005 and 31 December 2013 (Fig 1). Of these, 5,887 (0.9%) linked records were excluded because the dispensing and health data obviously did not refer to the same person and a further 267,070 people were excluded because they received simvastatin in 2005. Overall, there were 334,710 new users of simvastatin who had no history of a hospital admission with a renal condition before cohort entry (cohort 1) and 5,437 who had a history of non-dialysis dependent chronic kidney disease before cohort entry (cohort 2).

A total of 934 cases were identified from cohort 1 and 166 from cohort 2 (Fig 1). Controls could not be found for three cases in cohort 1 and six in cohort 2; hence the case-control analyses nested in cohorts 1 and 2 were based on 931 cases and 9299 controls, and 160 cases and 1084 controls, respectively. The characteristics of cases and controls selected from each cohort are shown in Table 1 (a longer version of the Table, which provides values for all variables included in the conditional logistic regression models, can be found in S4 Appendix). The median age at cohort entry of cases and controls selected from cohort 1 was 70 years and 42.3% were female; cases and controls from cohort 2 were about six years older and 35.6% and 27.3%, respectively, were female (the difference in proportions by sex was because, on average, female cases had fewer matched controls than male cases). The median time between cohort entry and the index date was 2.9 years for cases and controls from cohort 1 and about one year less for cases and controls from cohort 2. Cases from both cohorts were more likely than controls to be Māori (the indigenous people of New Zealand); belong to the most deprived socio-economic quintile; have a higher Charlson comorbidity score at cohort entry; and to have had a hospital discharge diagnosis before cohort entry of a cardiovascular condition and chronic liver disease; at least one cancer diagnosis in the Cancer Registry before cohort entry; a record of obesity or tobacco use at any time before the index date; and in the 90 days before that date to have had a hospital discharge diagnosis of acute myocardial infarction, congestive heart failure, exposure to intravenous radio-contrast, and sepsis. Cases from both cohorts were less likely than controls to have had a hospital discharge diagnosis of diabetes mellitus at any time before the index date. In the 90 days before the index date, cases from both cohorts were more likely than controls to have received many of the drugs known or suspected to cause AKI and/or to interact with statins (Table 2).

**Table 1:** Characteristics of cases and controls according to history of renal disease at cohort entry (simvastatin initiation). Values are numbers (percentages) unless stated otherwise.

Characteristic	Cases and controls selected from cohort 1		Cases and controls selected from cohort 2	
	Cases (n = 931)	Controls (n = 9299)	Cases (n = 160)	Controls (n = 1084)
**Median age at cohort entry (years, IQR)**	70 (61–78)	70 (61–78)	76 (67–82)	76 (69–81)
**Median follow-up from cohort entry to index date (years, IQR)**	2.9 (1.4–4.4)	2.9 (1.4–4.4)	1.9 (0.4–3.4)	2.0 (0.6–3.4)
**Female sex**	394 (42.3)	3940 (42.4)	57 (35.6)	296 (27.3)
**Ethnicity, prioritised**				
Māori	134 (14.4)	576 (6.2)	31 (19.4)	114 (10.5)
Pacific	43 (4.6)	420 (4.5)	2 (1.3)	113 (10.4)
Chinese	9 (1.0)	218 (2.3)	2 (1.3)	11 (1.0)
Other Asian	15 (1.6)	346 (3.7)	3 (1.9)	28 (2.6)
New Zealand European and Other	713 (76.6)	7137 (76.8)	119 (74.4)	796 (73.4)
Missing	17 (1.8)	602 (6.5)	3 (1.9)	22 (2.0)
**NZ Dep06 quintile**				
1 (least disadvantaged)	118 (12.7)	1532 (16.5)	27 (16.9)	112 (10.3)
2	136 (14.6)	1555 (16.7)	14 (8.8)	159 (14.7)
3	171 (18.4)	2065 (22.2)	28 (17.5)	209 (19.3)
4	238 (25.6)	2244 (24.1)	44 (27.5)	319 (29.4)
5 (most disadvantaged)	268 (28.8)	1870 (20.1)	47 (29.4)	280 (25.8)
Missing	0	33 (0.4)	0	5 (0.5)
**Charlson comorbidity score at cohort entry**				
0	292 (31.4)	6127 (65.9)	8 (5.0)	137 (12.6)
1	201 (21.6)	1607 (17.3)	15 (9.4)	(14.8)
> 1	438 (47.0)	1565 (16.8)	137 (85.6)	787 (72.6)
**Hospital discharge diagnoses at any time before cohort entry**				
Raised blood pressure	305 (32.8)	1685 (18.1)	127 (79.4)	702 (64.8)
Any ischaemic heart disease	157 (16.9)	1115 (12.0)	82 (51.3)	464 (42.8)
Acute myocardial infarction	85 (9.1)	552 (5.9)	48 (30.0)	255 (23.5)
Angina	54 (5.8)	478 (5.1)	38 (23.8)	202 (18.6)
Coronary artery bypass graft	9 (1.0)	76 (0.8)	12 (7.5)	59 (5.4)
Atrial fibrillation	98 (10.5)	612 (6.6)	63 (39.4)	288 (26.6)
Congestive heart failure	97 (10.4)	320 (3.4)	70 (43.8)	302 (27.9)
Ischaemic stroke	35 (3.8)	277 (3.0)	19 (11.9)	80 (7.4)
Other cerebrovascular disease	18 (1.9)	65 (0.7)	6 (3.8)	26 (2.4)
Peripheral arterial disease	38 (4.1)	169 (1.8)	20 (12.5)	114 (10.5)
Dyslipidaemia	63 (6.8)	480 (5.2)	30 (18.8)	243 (22.4)
Chronic liver disease	12 (1.3)	31 (0.3)	5 (3.1)	19 (1.8)
**Cancer registration at any time before cohort entry**	154 (16.5)	934 (10.0)	32 (20.0)	197 (18.2)
**Recorded history at any time before index date**				
Tobacco use^a^	251 (27.0)	1173 (12.6)	33 (20.6)	240 (22.1)
Obesity^b^	74 (7.9)	250 (2.7)	28 (17.5)	141 (13.0)
Diabetes mellitus^c^	138 (14.8)	1675 (18.0)	32 (20.0)	414 (38.2)
**Acute events and interventions in 90 days before index date (hospital discharge diagnoses)**^d^				
Acute myocardial infarction	48 (5.2)	71 (0.8)	14 (8.8)	39 (3.6)
Congestive heart failure	83 (8.9)	74 (0.8)	29 (18.1)	41 (3.8)
Exposure to intravenous radio-contrast	75 (8.1)	120 (1.3)	7 (4.4)	28 (2.6)
Sepsis	33 (3.5)	23 (0.2)	6 (3.8)	10 (0.9)
Chemotherapy	9 (1.0)	2 (0.02)	1 (0.6)	0

^a^ Hospital discharge diagnosis of tobacco use at any time before index date and/or dispensed smoking cessation pharmaceutical products in year before index date.

^b^ Hospital discharge diagnosis of obesity at any time before index date and/or dispensed weight loss pharmaceutical products in year before index date.

^c^ Hospital discharge diagnosis of diabetes mellitus (any type) at any time before index date and/or dispensed insulin and/or oral hypoglycaemic agents in year before index date.

^d^ In the 90 days before the index date 6 cases and 8 controls from cohort 1 and 6 controls from cohort 2 had a coronary artery bypass graft; 1 case and 3 controls from cohort 1 were diagnosed with shock. No cases or controls were diagnosed with major trauma, and no additional cases or controls had major surgery.

**Table 2:** Other prescription drugs dispensed in the 90 days before the index date.

Prescription drug dispensed^a^	Cases and controls selected from Cohort 1		Cases and controls selected from Cohort 2	
	Cases (n = 931)	Controls (n = 9299)	Cases (n = 160)	Controls (n = 1084)
Acyclovir	15 (1.6)	46 (0.5)	3 (1.9)	3 (0.3)
Amiodarone	23 (2.5)	75 (0.8)	5 (3.1)	25 (2.3)
Amphotericin	6 (0.6)	11 (0.1)	1 (0.6)	0
Angiotensin converting enzyme inhibitors	540 (58.0)	3517 (37.8)	103 (64.4)	544 (50.2)
Angiotensin receptor blockers	101 (10.8)	665 (7.2)	12 (7.5)	90 (8.3)
Azol antifungals	16 (1.7)	66 (0.7)	2 (1.3)	11 (1.0)
β-lactam antibiotics	142 (15.3)	846 (9.1)	43 (26.9)	124 (11.4)
Calcium channel blockers	265 (28.5)	2023 (21.8)	64 (40.0)	353 (32.6)
Diuretics	445 (47.8)	1983 (21.3)	119 (74.4)	495 (45.7)
Fibrates	26 (2.8)	133 (1.4)	2 (1.3)	15 (1.4)
Fusidic acid	17 (1.8)	146 (1.6)	3 (1.9)	20 (1.8)
Insulin	23 (2.5)	241 (2.6)	8 (5.0)	132 (12.2)
Macrolide antibiotics	73 (7.8)	337 (3.6)	18 (11.3)	61 (5.6)
Non-steroidal anti-inflammatories	151 (16.2)	867 (9.3)	7 (4.4)	78 (7.2)
Oral hypoglycaemic drugs	80 (8.6)	1134 (12.2)	11 (6.9)	171 (15.8)
Sulphonamides	57 (6.1)	68 (0.7)	12 (7.5)	16 (1.5)
Warfarin	110 (11.8)	517 (5.6)	26 (16.3)	147 (13.6)

^a^ In the 90 days before the index date 3 controls in cohort 1 and 1 control from cohort 2 were dispensed aminoglycosides, 12 cases from cohort 1 were dispensed cisplatin, 1 case and 2 controls from cohort 1 and 1 case and 8 controls from cohort 2 were dispensed cyclosporin, no cases or controls were dispensed danazol, 9 cases and 55 controls from cohort 1 and 1 case and 6 controls from cohort 2 were dispensed methotrexate, no cases or controls were dispensed nefazodone, 1 case and 13 controls from cohort 1 and 1 control from cohort 2 were dispensed nicotinic acid, no cases or controls were dispensed protease inhibitors, 1 case and 2 controls from cohort 1 and 3 controls from cohort 2 were dispensed tacrolimus, and 1 case and 20 controls from cohort 1 and 1 case and 3 controls from cohort 2 were dispensed thiazolidinediones/glitazones.

The results of the primary analysis are shown in Table 3. In the analysis based on patients with no history of a renal condition before cohort entry, there was a suggestion of a dose-response relationship in the unadjusted matched analysis; relative to current users of 20mg, users of 40mg and 80mg of simvastatin were 1.4 (95% CI 1.2–1.7, p < 0.0005) and 1.7 (95% CI 1.0–2.7, p = 0.03) times as likely to develop AKI without serious muscle injury, while recent users were 1.4 (95% CI 1.0–2.0, p = 0.04) times as likely. However, no such relationship was evident in the adjusted analysis. The numbers of cases and controls taking 60mg simvastatin were low and the confidence intervals in the unadjusted and adjusted analyses were correspondingly wide. In the primary analysis based on patients with a history of non-dialysis dependent chronic kidney disease before cohort entry, similar proportions of cases and controls were taking 40mg of simvastatin and there was no evidence of a dose-response relationship in the unadjusted or adjusted matched analyses. The proportions of cases and controls taking 80mg were also similar, but very small, and the 95% CI became very wide when we attempted to adjust adequately for confounding. In this analysis, past users were twice as likely to be admitted with a principal diagnosis of AKI without serious muscle injury when compared with current users of 20mg simvastatin (OR 2.0 [95% CI 1.1–3.8], p = 0.03); of these past users, 15 (32.6%) cases and 55 (22.4%) controls were dispensed atorvastatin, and no cases and two (0.8%) controls were dispensed pravastatin in the 90 days before the index date.

**Table 3:** Risk of acute kidney injury (without concurrent serious muscle injury) in relation to daily dose of simvastatin on the index date in cases and controls with no renal history, and in those with non-dialysis dependent chronic kidney disease, before first simvastatin dispensing.

	Cases (no [%])	Controls (no [%])	Matched odds ratios (95% CI)	p-value	Adjusted matched odds ratios^a^ (95% CI)	p-value
**No renal history**	**(n = 931)**	**(n = 9276)**^b^				
Current user of simvastatin						
10 mg	54 (5.8)	621 (6.7)	1.0 (0.7–1.3)	0.9	1.2 (0.9–1.8)	0.3
20 mg	229 (24.6)	2609 (28.1)	1.0 (reference)	–	1.0 (reference)	–
40 mg	248 (26.6)	2013 (21.7)	1.4 (1.2–1.7)	<0.0005	0.9 (0.7–1.2)	0.5
60 mg	2 (0.2)	39 (0.4)	0.6 (0.1–2.5)	0.5	0.4 (0.1–2.2)	0.3
80 mg	21 (2.3)	142 (1.5)	1.7 (1.0–2.7)	0.03	1.3 (0.7–2.3)	0.4
Recent user of simvastatin	48 (5.2)	385 (4.2)	1.4 (1.0–2.0)	0.04	1.4 (0.9–2.0)	0.1
Past user of simvastatin	329 (35.3)	3467 (37.4)	1.1 (0.9–1.3)	0.4	1.1 (0.9–1.4)	0.2
**Non-dialysis dependent chronic kidney disease**	**(n = 160)**	**(n = 1076)**^c^				
Current user of simvastatin						
10 mg	8 (5.0)	89 (8.3)	0.7 (0.3–1.5)	0.3	0.9 (0.3–2.3)	0.8
20 mg	45 (28.1)	319 (29.6)	1.0 (reference)	–	1.0 (reference)	–
40 mg	54 (33.8)	351 (32.6)	1.1 (0.7–1.8)	0.6	1.1 (0.7–1.9)	0.7
80 mg	2 (1.3)	21 (2.0)	0.8 (0.2–3.6)	0.8	2.0 (0.4–10.9)	0.4
Recent user of simvastatin	5 (3.1)	50 (4.6)	0.7 (0.3–2.0)	0.6	0.9 (0.3–3.1)	0.9
Past user of simvastatin	46 (28.8)	246 (22.9)	1.4 (0.8–2.2)	0.2	2.0 (1.1–3.8)	0.03

^a^ Confounders which were evaluated in the model are listed in the Methods section. The final parsimonious model for the analysis nested in cohort 1 included ethnicity; NZDep06; Charlson co-morbidity score; smoking (ever); diabetes mellitus (any type, ever); age at cohort entry; hospital discharge diagnosis of raised blood pressure, angina, other cerebrovascular disease, chronic liver disease before cohort entry; cancer registration before cohort entry; hospital discharge diagnosis of raised blood pressure, any ischaemic heart disease, any myocardial infarction, ischaemic stroke, renal disease between cohort entry and the index date; cancer registration between cohort entry and the index date; number of hospital admissions for any reason in the year before the index date; hospital discharge diagnosis of acute myocardial infarction, congestive heart failure, exposure to intravenous radio-contrast, sepsis in the 90 days before the index date; dispensed amphotericin, ACE inhibitors, amiodarone, angiotensin receptor blockers, β-lactam antibiotics, calcium channel blockers, diuretics, fibrates, macrolide antibiotics, NSAIDs, sulphonamides, warfarin in the 90 days before the index date. The final parsimonious model for the analysis nested in cohort 2 included ethnicity; NZDep06; Charlson co-morbidity score; smoking (ever); diabetes mellitus (any type, ever); hospital discharge diagnosis of raised blood pressure, any myocardial infarction, atrial fibrillation, dyslipidaemia, chronic liver disease before cohort entry; cancer registration before cohort entry; number of hospital admissions for any reason in the year before cohort entry; hospital discharge diagnosis of raised blood pressure, any ischaemic heart disease, atrial fibrillation, congestive heart failure, ischaemic stroke, renal disease between cohort entry and the index date; number of hospital admissions for any reason in the year before the index date; congestive heart failure in the 90 days before the index date; dispensed acyclovir, ACE inhibitors, β-lactam antibiotics, diuretics, macrolide antibiotics, NSAIDs, sulphonamides, warfarin in the 90 days before the index date.

^b^ 23 controls, but no cases, were current users of other daily doses of simvastatin (6 on 5mg, 1 on 15mg, 12 on 30mg, 2 on 50mg, and 2 on 120mg) and have been excluded from the analysis.

^c^ 8 controls, but no cases, were current users of other daily doses of simvastatin (1 on 30mg, and 7 on 60mg) and have been excluded from the analysis.

Tables 4 and 5 show the results of the secondary analyses. No relationship between simvastatin dose and AKI without serious muscle injury was found in the unadjusted or adjusted analyses restricted to the cases from cohort 1 who required dialysis (n = 63, 6.8%) and their controls (Table 4). It was not possible to undertake the corresponding analysis based on cases and controls from cohort 2 as the number of cases who received dialysis was very small (n = 18, 11.3%). The results of the fixed exposure analysis were broadly similar to those of the primary analysis (Table 5). In the analysis based on cases and controls with no history of a renal condition before cohort entry, there appeared to be a dose response relationship in the unadjusted matched analysis; however, this disappeared in the adjusted analysis. No dose-response relationship was found in the unadjusted or adjusted analyses based on cases and controls with a history of non-dialysis-dependent chronic kidney disease before cohort entry.

**Table 4:** Risk of acute kidney injury (without concurrent serious muscle injury) requiring dialysis in relation to daily dose of simvastatin on the index date in cases and controls with no renal history before first simvastatin dispensing.

Exposure status	Cases (no [%]) (n = 63)	Controls (no [%]) (n = 625)^a^	Matched odds ratios (95% CI)	p-value	Adjusted matched odds ratios^b^ (95% CI)	p-value
Current user of simvastatin						
10 mg	4 (6.3)	38 (6.1)	0.7 (0.2–2.1)	0.5	1.0 (0.3–3.4)	0.9
20 mg	26 (41.3)	175 (28.0)	1.0 (reference)	–	1.0 (reference)	–
40 mg	12 (19.0)	145 (23.2)	0.6 (0.3–1.2)	0.1	0.5 (0.2–1.3)	0.2
80 mg	5 (7.9)	16 (2.6)	2.2 (0.7–6.8)	0.2	2.2 (0.4–11.2)	0.3
Recent user of simvastatin	3 (4.8)	24 (3.8)	0.8 (0.2–2.9)	0.7	0.6 (0.1–2.9)	0.5
Past user of simvastatin	13 (20.6)	227 (36.3)	0.4 (0.2–0.8)	0.007	0.4 (0.2–1.0)	0.05

^a^ 5 controls, but no cases, were current users of other daily doses of simvastatin (1 on 5mg, and 4 on 60mg) and have been excluded from the analysis.

^b^ Confounders which were evaluated in the model are listed in the Methods section. The final parsimonious model included ethnicity; NZDep06; Charlson co-morbidity score; smoking (ever); diabetes mellitus (any type, ever); hospital discharge diagnosis of ischaemic heart disease, any myocardial infarction, coronary artery bypass graft before cohort entry; cancer registration before cohort entry; hospital discharge diagnosis of any ischaemic heart disease, angina, atrial fibrillation, congestive heart failure, renal disease between cohort entry and the index date; exposure to intravenous radio-contrast in the 90 days before the index date; dispensed β-lactam antibiotics, calcium channel blockers, diuretics, macrolide antibiotics, NSAIDs, warfarin in the 90 days before the index date.

**Table 5:** Risk of acute kidney injury (without concurrent serious muscle injury) in relation to daily dose of simvastatin dispensed at cohort entry in cases and controls with no renal history, and in those with non-dialysis dependent chronic kidney disease, before first simvastatin dispensing.

	Cases (no [%])	Controls (no [%])	Matched odds ratios (95% CI)	p-value	Adjusted matched odds ratios^a^ (95% CI)	p-value
**No renal history**	**(n = 930**)^b^	**(n = 9255)**^c^				
Current user of simvastatin						
10 mg	123 (13.2)	1371 (14.8)	1.0 (0.8–1.3)	0.8	1.1 (0.9–1.4)	0.4
20 mg	435 (46.8)	5018 (54.2)	1.0 (reference)	–	1.0 (reference)	–
40 mg	358 (38.5)	2802 (30.3)	1.5 (1.3–1.7)	< 0.0005	1.1 (0.9–1.3)	0.2
80 mg	14 (1.5)	64 (0.7)	2.5 (1.4–4.6)	0.002	1.5 (0.7–3.1)	0.3
**Non-dialysis dependent chronic kidney disease**	**(n = 160)**	**(n = 1073**)^d^				
Current user of simvastatin						
10 mg	15 (9.4)	140 (13.0)	0.7 (0.4–1.3)	0.2	0.7 (0.3–1.5)	0.4
20 mg	77 (48.1)	491 (45.8)	1.0 (reference)	–	1.0 (reference)	–
40 mg	64 (40.0)	435 (40.5)	1.0 (0.7–1.4)	0.9	1.0 (0.6–1.5)	0.9
80 mg	4 (2.5)	7 (0.7)	3.6 (0.9–13.7)	0.06	3.3 (0.5–21.6)	0.2

^a^ Confounders which were evaluated in the model are listed in the Methods section. The final parsimonious model for the analysis nested in cohort 1 included ethnicity; NZDep06; Charlson co-morbidity score; smoking (ever); diabetes mellitus (any type, ever); age at cohort entry; hospital discharge diagnosis of raised blood pressure, any ischaemic heart disease, angina, congestive heart failure, other cerebrovascular disease, chronic liver disease before cohort entry; cancer registration before cohort entry; hospital discharge diagnosis of raised blood pressure, any ischaemic heart disease, any myocardial infarction, ischaemic stroke, renal disease between cohort entry and index date; cancer registration between cohort entry and index date; number of hospital admissions for any reason in the year before index date; any acute myocardial infarction, congestive heart failure, exposure to intravenous radio-contrast, sepsis, chemotherapy in the 90 days before index date; dispensed amiodarone, amphotericin, ACE inhibitors, angiotensin receptor blockers, β-lactam antibiotics, calcium channel blockers, diuretics, fibrates, macrolide antibiotics, NSAIDs, sulphonamides, warfarin in the 90 days before index date. The final parsimonious model for the analysis nested in cohort 2 included ethnicity; NZDep06; Charlson co-morbidity score; smoking (ever); diabetes mellitus (ever, any type); hospital discharge diagnosis of raised blood pressure, any ischaemic heart disease, any myocardial infarction, atrial fibrillation, dyslipidaemia, chronic liver disease before cohort entry; cancer registration before cohort entry; number of hospital admissions for any reason in the year before cohort entry; hospital discharge diagnosis of raised blood pressure, any ischaemic heart disease, atrial fibrillation, congestive heart failure, ischaemic stroke, renal disease between cohort entry and index date; number of hospital admissions for any reason in the year before the index date; hospital discharge diagnosis of congestive heart failure in the 90 days before the index date; dispensed acyclovir, ACE inhibitors, β-lactam antibiotics, diuretics, sulphonamides, warfarin in the 90 days before index date.

^b^ 1 case was a user of 5mg of simvastatin daily and has been excluded from the analysis.

^c^ 44 controls were current users of other daily doses of simvastatin (9 on 5mg, 1 on 15mg, 16 on 30mg, 1 on 50mg, 15 on 60mg, and 2 on 120mg) and have been excluded from the analysis.

^d^ 11 controls, and no cases, were current users of other daily doses of simvastatin (1 on 5 mg, 1 on 30mg, 7 on 60mg, 1 on 120mg, and 1 on 160 mg) and have been excluded from the analysis.

Some of the cases and controls selected from cohort 1 (175 [18.8%] cases, 198 [2.1%] controls) and cohort 2 (45 [28.1%] cases, 109 [10.1%] controls) were discharged from hospital at least once between cohort entry and their index date with an additional (i.e. not principal) diagnosis of N17. We undertook two sensitivity analyses to explore whether this might have had an impact on the results of the primary analysis. First, we excluded all cases and controls with an additional N17 diagnosis from the analysis. Second, in a worst-case scenario, we assumed that patients who changed dose or stopped simvastatin immediately following the N17 admission had continued on the dose they were taking before the admission. The results of these analyses did not differ appreciably from those of the primary analyses (data not shown).

There were also some cases and controls who were classified as current users of simvastatin who had switched from simvastatin to either atorvastatin or pravastatin in the 30 day period before the index date; in cohort 1, nine cases and 47 controls switched to atorvastatin and one control switched to pravastatin. In cohort 2, two cases and three controls switched to atorvastatin and one control switched to pravastatin. We undertook a third sensitivity analysis excluding these cases and controls; this did not alter our conclusions (data not shown).

Overall, there were 557 cases (including the three for whom no controls were found) in cohort 1 who were taking simvastatin at the time they became cases and 818,445 person-years of simvastatin exposure; hence the incidence rate of AKI without serious muscle injury in patients with no history of a renal condition was 6.8 (95% CI 6.3–7.4) per 10,000 person-years of simvastatin use. The rate in simvastatin users with a history of non-dialysis dependent chronic kidney disease was much higher at 88 (95% CI 73–105) per 10,000 person-years (based on 112 cases, including three of six for whom no controls were found, and 12,744 person-years of simvastatin exposure).

Three (0.5%) of the 557 cases in cohort 1 and none of 112 cases in cohort 2 who were current users of simvastatin had been reported to CARM. There were no cases in the CARM database who met our eligibility criteria but were not identified and included in our study.

## Discussion

### Principal findings

In this large nationwide study of new users of simvastatin we found no evidence of a relationship between simvastatin dose and the occurrence of AKI without serious muscle injury in two cohorts of patients: those with no hospital admissions with a renal condition before starting simvastatin and those who had been admitted with non-dialysis dependent chronic kidney disease before initiating simvastatin use. Nor did we find a dose-response relationship in a secondary analysis confined to cases who required dialysis, or in a fixed exposure analysis based on the simvastatin dose cases and controls first received.

### Strengths and limitations

This investigation has several strengths and limitations which require discussion. First, we are likely to have picked up all simvastatin dispensings in New Zealand as the Pharmaceutical Collection contains records of all publicly funded dispensings from all community pharmacies and pharmacists are not reimbursed unless they submit a claim. Furthermore, almost all simvastatin dispensing records during the study period contained an NHI which meant we were able to identify virtually all users of simvastatin and link to information about their use of other drugs, as well as demographic data, details of any hospital admissions, cancer registrations, and death records. Second, we are likely to have identified all cohort members who were admitted to hospital with AKI during the study period as public and private hospitals are required to report all inpatient and day patient discharges; similarly, all deaths (in hospital or in the community) will have been identified as death certification is a legal requirement. Moreover, the coding of hospital discharge diagnoses and causes of death is undertaken by professional nosologists. Third, there is no unique rubric for rhabdomyolysis in ICD-10-AM so in order to exclude people who developed AKI as a consequence of muscle injury we excluded patients with a principal diagnosis of N17 and at least one of several concurrent muscle-related diagnoses. While it is possible we might have excluded some genuine cases, large numbers on higher doses of simvastatin would need to have been unnecessarily excluded to account for the absence of a relationship between simvastatin dose and AKI without serious muscle injury. Reassuringly, our review of CARM records did not identify any eligible cases that we had failed to include. Fourth, we did not have access to information about changes in baseline serum creatinine and urine output in order to validate the diagnosis of AKI [44]; consequently it is possible that there was some misclassification of case status. However, cases were required to have a principal, not additional, diagnosis of N17 which makes it less likely that we included patients with milder renal impairment. Moreover, the findings of the secondary analysis confined to cases who required dialysis were consistent with those of the primary analysis. Fifth, we were unable to find controls for three cases in cohort 1 and six in cohort 2, but the exclusion of such a small number of cases is insufficient to explain our null findings. Sixth, controls were matched to cases by age, sex, and cohort entry date, and we explored potential confounding by a large number of potential risk factors for AKI in matched analyses. Most drugs known or suspected to increase the risk of AKI are prescription-only and publicly funded, therefore their dispensing from community pharmacies should have appeared in the Pharmaceutical Collection. It is possible, however, that the dispensing of some drugs (e.g. short courses of certain antibiotics in earlier years of the study period) could have been missed as a result of the cost of the drug being less than the patient co-payment. In addition, we did not have information about the use of over-the-counter drugs. However, while NSAIDs (an important cause of AKI) are available over-the-counter, most chronic users will receive these via prescription, because of the lower cost. Inpatient use of drugs is also not recorded in the Pharmaceutical Collection, although we were able to identify patients who were admitted to hospital shortly before the index date. Because of the very large number of procedure codes recorded in the ACHI, we followed the advice of a professional clinical coder and used general and spinal anaesthesia codes (excluding spinal anaesthesia in childbirth) to identify major surgery. It is possible that by following this approach we might have missed some surgical interventions and there might have been some residual confounding; however, to explain our null findings, we would need to have missed surgery in a substantial number of patients taking 20mg simvastatin and there is no reason to suppose that patients on lower doses of simvastatin are more likely to undergo surgery than those on higher doses. We were unable to adjust for comorbidities which did not result in hospital admission or the use of prescription medicines, and this might have led to incomplete control of confounding. Seventh, the numbers of cases and controls from cohort 2 who were taking 80mg simvastatin were very small, limiting our ability to examine the risk associated with this dose. Eighth, the explanation for the significantly higher risk of AKI without serious muscle injury in past users of simvastatin with non-dialysis dependent chronic kidney disease is unclear; one possibility is that sicker patients were more likely to discontinue statin therapy. Finally, our data only related to simvastatin, which until December 2013 was recommended in New Zealand as the first-line statin for the primary and secondary prevention of cardiovascular events. However, the current guidelines still recommend simvastatin 40mg (or atorvastatin 20mg) for patients with a 5-year combined cardiovascular risk of 10–20% [33], so an investigation into the risk of AKI without serious muscle injury in simvastatin users was warranted.

### Comparison with previous studies

In contrast to the present investigation, four previous observational studies have reported an association between AKI and statin dose or potency, although a relationship was not consistently found in all patient groups or in all time periods following statin initiation, and none of the studies examined the risk of AKI in the absence of serious muscle injury [9–12]. In the first investigation, a cohort study based on general practice data, an apparent dose-response was reported in patients starting simvastatin; relative to non-users, the adjusted hazard ratio (HR) for patients taking 40 or 80mg of simvastatin daily (2.02 [95% CI 1.63–2.52]) was higher than the HR for patients taking 10 or 20mg daily (1.39 [95% CI 1.14–1.70]), although this could have been a chance finding [9]. Our analyses were based on substantially more simvastatin users and AKI cases and we were able to compare, directly, the risk of AKI without serious muscle injury in users of different doses of simvastatin. In the second study, a meta-analysis of nine nested case-control studies which had all followed a common protocol and had a maximum of two years’ follow-up, patients without a history of renal disease who received high potency statin treatment (defined as at least 40mg simvastatin, 20mg atorvastatin, or 10mg rosuvastatin) had a greater risk of hospital admission with AKI than patients receiving low potency therapy [10]. The adjusted rate ratio in the first 120 days of treatment was 1.34 (95% CI 1.25–1.43) and a significantly increased risk of AKI persisted throughout the first two years of use. Conversely, and in line with our findings, there appeared to be no association between statin potency and AKI in patients with a history of non-dialysis dependent chronic kidney disease. In the third investigation, a case-control study nested within a cohort of new users of statins with no admissions for renal disease in the previous seven years, users of high potency statins (defined as in the second study) [10] were more likely than users of low potency statins to have been admitted to hospital with AKI during the first six months of use (adjusted OR 1.54 [95% CI 1.25–1.91]); however, no significant association was found when the period of interest was extended to 12 months (adjusted OR 1.17 [95% CI 0.89–1.54]). Finally, a secondary analysis in a retrospective cohort study of statin users and non-users found that high intensity statin users (undefined) were 1.24 times as likely as low-to-moderate intensity statin users to be diagnosed with acute or unspecified renal failure during follow-up (OR 1.24 [95% CI 1.13–1.37]), although it is unclear whether potential confounders were taken into account in this particular analysis and no relationship was observed for a healthy sub-cohort without chronic renal disease [12].

## Conclusions

The biological mechanism behind the reported excess risk of AKI in users of higher versus lower dose or potency statins is unclear [6]. Our failure to find an increase in risk with higher doses of simvastatin after the systematic exclusion of patients with concomitant muscle diagnoses, and conversely our finding in an earlier study based on the same data sources of a relationship between simvastatin dose and the risk of rhabdomyolysis [20], suggests that a relationship between statin dose and AKI may not exist independent of serious muscle injury. Further research is required to confirm this finding, including investigations focussing on other statins.

## Supporting information

S1 Appendix International Statistical Classification of Diseases and Related Health Problems, Australian Modification (ICD-AM), and Australian Classification of Health Intervention (ACHI) rubrics used to identify admissions with renal conditions before cohort entry. (PDF)

S2 Appendix Identification of cohort members with a history of hospital admission with non-dialysis dependent chronic kidney disease before cohort entry. (PDF)

S3 Appendix International Statistical Classification of Diseases and Related Health Problems, Australian Modification tenth revision (ICD-AM-10), muscle-related rubrics under which rhabdomyolysis may be classified. (PDF)

S4 Appendix Supplemental Table 1. Characteristics of cases and controls according to history of renal disease at cohort entry (simvastatin initiation). Values are given for all variables included in the adjusted analyses and are numbers (percentages) unless stated otherwise.
(PDF)
