# Supplementary material for: Simvastatin dose and acute kidney injury without concurrent serious muscle injury: A nationwide nested case-control study
Source: PLoS One. 2017 Jul 28;12(7):e0182066. doi: 10.1371/journal.pone.0182066 (PMC5533333; doi:10.1371/journal.pone.0182066)
Supplement: S1 Appendix — (PDF) [file pone.0182066.s001.pdf]

S1 Appendix 1. International Statistical Classification of Diseases and Related Health Problems, Australian Modification (ICD-AM), and Australian Classification of Health Intervention (ACHI) rubrics used to identify admissions with renal conditions before cohort entry.

(a) ICD-9-AM rubrics (for discharges before ICD-10-AM introduced in 1999)

**Description**	**ICD-9-AM**
Acute glomerulonephritis	580
Nephrotic syndrome	581
Chronic glomerulonephritis	582
Nephritis and nephropathy, not specified as acute or chronic	583
Acute renal failure	584
Chronic kidney disease	585
Renal failure, unspecified	586
Renal sclerosis, unspecified	587
Disorders resulting from impaired renal function	588
Chronic pyelonephritis	5900
Acute pyelonephritis	5901
Renal and perinephric abscess	5902
Other pyelonephritis or pyonephrosis, not specified as acute or chronic	5908
Infection of kidney, unspecified	5909
Hydronephrosis	591
Vesicoureteral reflux	5937
Hypertension secondary to renal disease complicating pregnancy childbirth and the puerperium	6421
Renal dialysis status	V451
Encounter for dialysis and dialysis catheter care	V56
Arteriovenostomy for renal dialysis	3927 (procedure code)
Haemodialysis	3995 (procedure code)
Peritoneal dialysis	5498 (procedure code)

(b) ICD-10-AM rubrics

Description	ICD-10-AM
Acute nephritic syndrome	N00
Rapidly progressive nephritic syndrome	N01
Recurrent and persistent haematuria	N02
Chronic nephritic syndrome	N03
Nephrotic syndrome	N04
Unspecified nephritic syndrome	N05
Isolated proteinuria with specified morphological lesion	N06
Hereditary nephropathy, not elsewhere classified	N07
Glomerular disorders in diseases classified elsewhere	N08
Acute tubulo-interstitial nephritis	N10
Chronic tubulo-interstitial nephritis	N11
Tubulo-interstitial nephritis, not specified as acute or chronic	N12
Obstructive and reflux uropathy	N13
Drug- and heavy-metal-induced tubulo-interstitial and tubular conditions	N14
Other renal tubulo-interstitial diseases	N15
Renal tubulo-interstitial disorders in diseases classified elsewhere	N16
Acute renal failure	N17
Chronic kidney disease	N18
Unspecified kidney failure	N19
Disorders resulting from impaired renal tubular function	N25
Hypertensive renal disease	I12
Hypertensive heart and renal disease	I13
Pre-existing hypertensive renal disease complicating pregnancy, childbirth and the puerperium (any condition in I12 specified as a reason for obstetric care during pregnancy, childbirth or the puerperium)	O102
Pre-existing hypertensive heart and renal disease complicating pregnancy, childbirth and the puerperium (any condition in I13 specified as a reason for obstetric care during pregnancy, childbirth or the puerperium)	O103
Care involving dialysis	Z49

Description	ICD-10-AM
Dependence on renal dialysis	Z992
Unintentional cut, puncture, perforation, or haemorrhage during kidney dialysis or other perfusion	Y602
Foreign object accidentally left in body during kidney dialysis or other perfusion	Y612
Failure of sterile precautions during kidney dialysis or other perfusion	Y622
Kidney dialysis as the cause of abnormal reaction of the patient, or of later complication, without mention of misadventure at the time of the procedure	Y841
Mechanical complication of vascular dialysis catheter	T824
Mechanical complication of intraperitoneal dialysis catheter	T856
**Description**	**Australian Classification of Health Interventions**
Haemodialysis	13100-00
Intermittent haemofiltration	13100-01
Continuous haemofiltration	13100-02
Intermittent haemodiafiltration	13100-03
Continuous haemodiafiltration	13100-04
Haemoperfusion	13100-05
Peritoneal dialysis, short term	13100-06
Intermittent peritoneal dialysis, long term	13100-07
Continuous peritoneal dialysis, long term	13100-08
Establishment of peritoneal dialysis by abdominal puncture and insertion of temporary catheter	13112-00
Removal of temporary catheter for peritoneal dialysis	90351-00
Insertion and fixation of indwelling peritoneal catheter for long term peritoneal dialysis	13109-00
Replacement of indwelling peritoneal catheter for peritoneal dialysis	13109-01
Removal of indwelling peritoneal catheter for peritoneal dialysis	13110-00
Education and training for home dialysis	13104-00
