# Supplementary material for: Simvastatin dose and acute kidney injury without concurrent serious muscle injury: A nationwide nested case-control study
Source: PLoS One. 2017 Jul 28;12(7):e0182066. doi: 10.1371/journal.pone.0182066 (PMC5533333; doi:10.1371/journal.pone.0182066)
Supplement: S2 Appendix — (PDF) [file pone.0182066.s002.pdf]

S2 Appendix 2. Identification of cohort members with a history of hospital admission with non-dialysis dependent chronic kidney disease before cohort entry.

Patients meeting either of the following criteria before cohort entry were classified as having a history of non-dialysis dependent chronic kidney disease:

Criterion 1 (based on ICD-9-AM rubrics for hospital admissions before the introduction of ICD-10-AM in 1999)

An admission before cohort entry with a principal or additional diagnosis of 585 (Chronic kidney disease) and no admissions before cohort entry with any of the following dialysis-related codes:

i. Diagnosis codes
   - V451 (Renal dialysis status)
   - V56 (Encounter for dialysis and dialysis catheter care)

ii. Procedure codes
   - 3927 (Arteriovenostomy for renal dialysis)
   - 3995 (Haemodialysis)
   - 5498 (Peritoneal dialysis)

Criterion 2 (based on ICD-10-AM rubrics):

An admission before cohort entry with a principal or additional diagnosis of N18 (Chronic kidney disease) and no admissions before cohort entry with any of the ICD-10-AM diagnostic and Australian Classification of Health Intervention dialysis-related codes listed below.

Description	ICD-10-AM
Care involving dialysis	Z49
Dependence on renal dialysis	Z992
Unintentional cut, puncture, perforation, or haemorrhage during kidney dialysis or other perfusion	Y602
Foreign object accidentally left in body during kidney dialysis or other perfusion	Y612
Failure of sterile precautions during kidney dialysis or other perfusion	Y622
Kidney dialysis as the cause of abnormal reaction of the patient, or of later complication, without mention of misadventure at the time of the procedure	Y841
Mechanical complication of vascular dialysis catheter	T824
Mechanical complication of intraperitoneal dialysis catheter	T856
**Description**	**Australian Classification of Health Interventions**
Haemodialysis	13100-00
Intermittent haemofiltration	13100-01
Continuous haemofiltration	13100-02
Intermittent haemodiafiltration	13100-03
Continuous haemodiafiltration	13100-04
Haemoperfusion	13100-05
Peritoneal dialysis, short term	13100-06
Intermittent peritoneal dialysis, long term	13100-07
Continuous peritoneal dialysis, long term	13100-08
Establishment of peritoneal dialysis by abdominal puncture and insertion of temporary catheter	13112-00
Removal of temporary catheter for peritoneal dialysis	90351-00
Insertion and fixation of indwelling peritoneal catheter for long term peritoneal dialysis	13109-00
Replacement of indwelling peritoneal catheter for peritoneal dialysis	13109-01
Removal of indwelling peritoneal catheter for peritoneal dialysis	13110-00
Education and training for home dialysis	13104-00
