# Supplementary material for: Simvastatin dose and acute kidney injury without concurrent serious muscle injury: A nationwide nested case-control study
Source: PLoS One. 2017 Jul 28;12(7):e0182066. doi: 10.1371/journal.pone.0182066 (PMC5533333; doi:10.1371/journal.pone.0182066)
Supplement: S3 Appendix — (PDF) [file pone.0182066.s003.pdf]

S3 Appendix 3. International Statistical Classification of Diseases and Related Health Problems, Australian Modification tenth revision (ICD-AM-10), muscle-related rubrics under which rhabdomyolysis may be classified.

**Description**	**ICD-10-AM**
Other myopathies	G72
Other specified disorders of muscle	M62.8
Disorder of muscle, unspecified	M62.9
Myoglobinuria	R82.1
Traumatic ischaemia of muscle	T79.6
