# Supplementary material for: Simvastatin dose and acute kidney injury without concurrent serious muscle injury: A nationwide nested case-control study
Source: PLoS One. 2017 Jul 28;12(7):e0182066. doi: 10.1371/journal.pone.0182066 (PMC5533333; doi:10.1371/journal.pone.0182066)
Supplement: S4 Appendix — Characteristics of cases and controls according to history of renal disease at cohort entry (simvastatin initiation). Values are given for all variables included in the adjusted analyses and are numbers (percentages) unless stated otherwise. (PDF) [file pone.0182066.s004.pdf]

S4 Appendix 4. Supplemental Table 1. Characteristics of cases and controls according to history of renal disease at cohort entry (simvastatin initiation). Values are given for all variables included in the adjusted analyses and are numbers (percentages) unless stated otherwise.

**Characteristic**	**Cases and controls selected from cohort 1**		**Cases and controls selected from cohort 2**	
	**Cases (n=931)**	**Controls (n=9299)**	**Cases (n=160)**	**Controls (n=1084)**
**Median age at cohort entry (years, IQR)**	70 (61–78)	70 (61–78)	76 (67–82)	76 (69–81)
**Median follow-up from cohort entry to index date (years, IQR)**	2.9 (1.4–4.4)	2.9 (1.4–4.4)	1.9 (0.4–3.4)	2.0 (0.6–3.4)
**Female sex**	394 (42.3)	3940 (42.4)	57 (35.6)	296 (27.3)
**Ethnicity, prioritised**				
Māori	134 (14.4)	576 (6.2)	31 (19.4)	114 (10.5)
Pacific	43 (4.6)	420 (4.5)	2 (1.3)	113 (10.4)
Chinese	9 (1.0)	218 (2.3)	2 (1.3)	11 (1.0)
Other Asian	15 (1.6)	346 (3.7)	3 (1.9)	28 (2.6)
New Zealand European and Other	713 (76.6)	7137 (76.8)	119 (74.4)	796 (73.4)
Missing	17 (1.8)	602 (6.5)	3 (1.9)	22 (2.0)
**NZ Dep06 quintile**				
1 (least disadvantaged)	118 (12.7)	1532 (16.5)	27 (16.9)	112 (10.3)
2	136 (14.6)	1555 (16.7)	14 (8.8)	159 (14.7)
3	171 (18.4)	2065 (22.2)	28 (17.5)	209 (19.3)
4	238 (25.6)	2244 (24.1)	44 (27.5)	319 (29.4)
5 (most disadvantaged)	268 (28.8)	1870 (20.1)	47 (29.4)	280 (25.8)
Missing	0	33 (0.4)	0	5 (0.5)

**Charlson comorbidity score at cohort entry**				
0	292 (31.4)	6127 (65.9)	8 (5.0)	137 (12.6)
1	201 (21.6)	1607 (17.3)	15 (9.4)	160 (14.8)
2	201 (21.6)	922 (9.9)	30 (18.8)	175 (16.1)
3	117 (12.6)	363 (3.9)	39 (24.4)	246 (22.7)
4	70 (7.5)	166 (1.8)	32 (20.0)	166 (15.3)
5	29 (3.1)	81 (0.9)	19 (11.9)	111 (10.2)
6	17 (1.8)	25 (0.3)	10 (6.3)	53 (4.9)
7	2 (0.2)	6 (0.1)	7 (4.4)	32 (3.0)
8	1 (0.1)	2 (0.0)	0	2 (0.2)
9	1 (0.1)	0	0	2 (0.2)
**Recorded history at any time before index date**				
Tobacco use[a]	251 (27.0)	1173 (12.6)	33 (20.6)	240 (22.1)
Obesity[b]	74 (7.9)	250 (2.7)	28 (17.5)	141 (13.0)
Diabetes mellitus[c]	138 (14.8)	1675 (18.0)	32 (20.0)	414 (38.2)

**Hospital discharge diagnoses at any time before cohort entry**				
Raised blood pressure	305 (32.8)	1685 (18.1)	127 (79.4)	702 (64.8)
Any ischaemic heart disease	157 (16.9)	1115 (12.0)	82 (51.3)	464 (42.8)
Any myocardial infarction	95 (10.2)	636 (6.8)	61 (38.1)	308 (28.4)
Acute myocardial infarction	85 (9.1)	552 (5.9)	48 (30.0)	255 (23.5)
Angina	54 (5.8)	478 (5.1)	38 (23.8)	202 (18.6)
Coronary artery bypass graft	9 (1.0)	76 (0.8)	12 (7.5)	59 (5.4)
Atrial fibrillation	98 (10.5)	612 (6.6)	63 (39.4)	288 (26.6)
Congestive heart failure	97 (10.4)	320 (3.4)	70 (43.8)	302 (27.9)
Ischaemic stroke	35 (3.8)	277 (3.0)	19 (11.9)	80 (7.4)
Other cerebrovascular disease	18 (1.9)	65 (0.7)	6 (3.8)	26 (2.4)
Peripheral arterial disease	38 (4.1)	169 (1.8)	20 (12.5)	114 (10.5)
Dyslipidaemia	63 (6.8)	480 (5.2)	30 (18.8)	243 (22.4)
Chronic liver disease	12 (1.3)	31 (0.3)	5 (3.1)	19 (1.8)
**Cancer registration at any time before cohort entry**	154 (16.5)	934 (10.0)	32 (20.0)	197 (18.2)
**Number of hospital admissions for any reason in year before cohort entry**				
None	571 (61.3)	6,930 (74.5)	49 (30.6)	406 (37.5)
1	233 (25.0)	1,726 (18.6)	53 (33.1)	360 (33.2)
2	77 (8.3)	432 (4.6)	31 (19.4)	178 (16.4)
≥ 3	50 (5.4)	211 (2.3)	27 (16.9)	140 (12.9)

**Hospital discharge diagnoses between cohort entry and index date**				
Raised blood pressure	315 (33.8)	1332 (14.3)	70 (43.8)	314 (29.0)
Any ischaemic heart disease	188 (20.2)	979 (10.5)	40 (25.0)	212 (19.6)
Any myocardial infarction	113 (12.1)	500 (5.4)	28 (17.5)	130 (12.0)
Acute myocardial infarction	104 (11.2)	412 (4.4)	25 (15.6)	110 (10.1)
Angina	68 (7.3)	457 (4.9)	17 (10.6)	95 (8.8)
Coronary artery bypass graft	21 (2.3)	119 (1.3)	0	12 (1.1)
Atrial fibrillation	156 (16.8)	551 (5.9)	29 (18.1)	135 (12.5)
Congestive heart failure	174 (18.7)	355 (3.8)	55 (34.4)	155 (14.3)
Ischaemic stroke	29 (3.1)	200 (2.2)	4 (2.5)	36 (3.3)
**Hospital discharge diagnoses between cohort entry and index date**				
Other cerebrovascular disease	16 (1.7)	72 (0.8)	3 (1.9)	10 (0.9)
Peripheral arterial disease	37 (4.0)	128 (1.4)	9 (5.6)	49 (4.5)
Dyslipidaemia	68 (7.3)	403 (4.3)	12 (7.5)	82 (7.6)
Chronic liver disease	9 (1.0)	14 (0.2)	3 (1.9)	7 (0.6)
Renal disease	179 (19.2)	292 (3.1)	75 (46.9)	275 (25.4)
**Cancer registration between cohort entry and index date**	195 (20.9)	465 (5.0)	13 (8.1)	60 (5.5)

**Number of hospital admissions for any reason in year before index date**				
None	317 (34.0)	6571 (70.7)	32 (20.0)	448 (41.3)
1	231 (24.8)	1688 (18.2)	41 (25.6)	279 (25.7)
2	152 (16.3)	603 (6.5)	34 (21.3)	163 (15.0)
≥ 3	231 (24.8)	437 (4.7)	53 (33.1)	194 (17.9)
**Acute events and interventions in 90 days before index date (hospital discharge diagnoses)**[d]				
Acute myocardial infarction	48 (5.2)	71 (0.8)	14 (8.8)	39 (3.6)
Congestive heart failure	83 (8.9)	74 (0.8)	29 (18.1)	41 (3.8)
Exposure to intravenous radio-contrast	75 (8.1)	120 (1.3)	7 (4.4)	28 (2.6)
Sepsis	33 (3.5)	23 (0.2)	6 (3.8)	10 (0.9)
Chemotherapy	9 (1.0)	2 (0.02)	1 (0.6)	0

[a] Hospital discharge diagnosis of tobacco use at any time before index date and/or dispensed smoking cessation pharmaceutical products in year before index date.

[b] Hospital discharge diagnosis of obesity at any time before index date and/or dispensed weight loss pharmaceutical products in year before index date.

[c] Hospital discharge diagnosis of diabetes mellitus (any type) at any time before index date and/or dispensed insulin and/or oral hypoglycaemic agents in year before index date.

[d] In the 90 days before the index date 6 cases and 8 controls from cohort 1 and 6 controls from cohort 2 had a coronary artery bypass graft; 1 case and 3 controls from cohort 1 were diagnosed with shock. No cases or controls were diagnosed with major trauma, and no additional cases or controls had major surgery.
